# Benchmarking Stability of Bipedal Locomotion Based on Individual Full Body Dynamics and Foot Placement Strategies–Application to Impaired and Unimpaired Walking

**DOI:** 10.3389/frobt.2018.00117

**Published:** 2018-10-12

**Authors:** Khai-Long Ho Hoang, Sebastian I. Wolf, Katja Mombaur

**Affiliations:** ^1^Optimization, Robotics and Biomechanics, Institute of Computer Engineering, Heidelberg University, Heidelberg, Germany; ^2^Clinic for Orthopedics and Trauma Surgery, Heidelberg University Hospital, Heidelberg, Germany

**Keywords:** benchmarking, capture point, foot placement, multibody dynamics, optimal control, stability, transfemoral prosthesis, walking

## Abstract

The principles underlying smooth and effortless human walking while maintaining stability as well as the ability to quickly respond to unexpected perturbations result from a plethora of well-balanced parameters, most of them yet to be determined. In this paper, we investigate criteria that may be useful for benchmarking stability properties of human walking. We perform dynamic reconstructions of human walking motions of unimpaired subjects and subjects walking with transfemoral prostheses from motion capture recordings using optimal control. We aim at revealing subject-specific strategies in applying dynamics in order to maintain steady gait considering irregularities such as deviating gait patterns or asymmetric body segment properties. We identify foot placement with respect to the Instantaneous Capture Point as the strategy globally applied by the subjects to obtain steady gait and propose the Residual Orbital Energy as a measure allowing for benchmarking human-like gait toward confident vs. cautious gait.

## 1. Introduction

Human and human-like walking motions form an important and challenging class of motions with respect to dynamics and control. The development of efficient measures for benchmarking bipedal locomotion is an important topic for many fields of research, ranging from human motion studies in biomechanics or medical fields to the development and control of humanoid robots, exoskeletons, prostheses etc. Among others, benchmarking allows to define walking standards, measure progress of human walking during therapy, define training goals or to compare different robot platforms, alternative prostheses models or tunings for a patient, different exoskeleton technologies or control algorithms, etc. Good benchmarking measures allow to shift from a purely qualitative comparison of motions to a more useful quantitative one that precisely assesses the quantities in a motion that are considered relevant for quality, thus giving helpful insights for improvement.

There are many different types of benchmarking measures. In the KoroiBot project, which studied human locomotion with the intention to improve walking qualities of humanoid robots, we have distinguished three different groups of key performance indicators (KPIs) to benchmark locomotion (see Schubert et al., 2014). The first group concerns technical indicators for walking performance which are equally important for all the fields discussed above and which have also been discussed in Torricelli et al. (2015). The second group concerns computational properties of the algorithms used for generating and controlling locomotion, so it is a criterion which is mainly relevant for technical systems, i.e., robots or controlled technical assistive devices. The third group contains high level KPIs and aims to asses to which amount motions of robots are human-like, a concept that can also be extended to investigate the effect of impairments or assistive devices to human walking motions. This evaluation is, of course, to some extent related to the technical performance evaluation on the lower level.

Technical performance indicators include elementary characteristics of walking performance such as walking speed in different walking scenarios, step length and step width, different measures for energy consumption, efficiency and cost of transport, as well as measures related to the walking scenario, such as manageable stair height, slope inclination or roughness of terrain. These criteria are straightforward to define and in most cases also quite easy to measure on a given system and scenario. Technical performance indicators however also include measures of stability and robustness of motions which are less straightforward to define and, as we will review in the following paragraph, for which no uniform consensus exists yet.

Maintaining stability and being robust also to larger perturbations that might occur, is one of the major objectives during a locomotion task. Human walking is characterized by a repetitive sequence of well-coordinated motions of the upper and lower limbs which carry the human body into a desired direction. The *stance* leg serves as a body support while the *swing* leg is moved toward the next support location. As described in Perry and Burnfield (2010), the legs alternate their roles in a reciprocal manner until the subject intents to stop. According to Winter (1995), due to the elevated center of mass (COM) balancing over the small contact surface established by the feet, the human body is an inherently unstable system for which stability is maintained by a continuously acting control system as well as by exploiting the whole-body dynamics. Stability of human walking describes the ability to maintain the intended locomotion task without falling.

The focus of this paper will be to evaluate several criteria for benchmarking stability of bipedal locomotion. We are particularly interested in criteria that can be applied to all fields of application listed above. As we will outline in the following paragraph, several approaches exist for controlling stability of humanoid robots - which, however, are not based on a criterion relevant for humans - and on the other side for evaluating perturbation reaction in human walking *a posteriori* which are unsuitable to predict or control behavior of robots.

### 1.1. Related research

An intuitive approach to bipedal locomotion defines any gait as stable as long as it does not lead to a fall. The set of all states of a walker which leads to stable gait according to this definition has been termed the *viability kernel* by Wieber (2002). Due to the vast computational effort required to compute this set as well as the lack of methods to generate control strategies based on this definition, the viability kernel has not yet found any practical application.

Stability in human walking has also been approached by Mombaur et al. (2001) by describing the human body as a hybrid dynamic system and examining its properties in terms of Lyapunov stability. This approach has led to some insights into the self-stabilization properties of human locomotion mechanics. However, to fully understand Lyapunov stability properties of human movement, fundamental knowledge about the feedback loops which are active during human locomotion would be required and would have to be included in the model, but it is not available yet. Modeling the human response to unpredictable changes in the environment in terms of a hybrid dynamic system has so far been an unsolved task (see Bruijn et al., 2013).

Other approaches which have been widely used in both clinical applications and the research community working on humanoid robotics derive control laws based on *ground reference points* which require a minimal amount of computational effort to be obtained and can be evaluated in real-time (Popovic et al., 2005). Other ground reference points which consider the velocities of the bipedal walker enable to explain foot placement and fall prevention as a response to sudden pushes Pratt and Tedrake (2006).

Maintaining the *Ground Center of Mass (GCoM)*, i.e., the projection of the Center of Mass on the ground plane, within the borders of the *Base of Support (BoS)*, i.e., the convex hull spanned by the contact points of the system with the ground, can be used as a very simple requirement for static stability of bipedal systems (Berns et al., 1994; Goswami, 1999). However, in our context, this approach is only applicable for static poses or quasi-static motions and is not feasible for describing the stability of dynamic bipedal motions such as human walking.

A very popular approach to stability in bipedal locomotion is based on the *Zero Moment Point (ZMP)*, introduced by Vukobratovic and Branislav (2004), which is defined as the ground reference point in which the resulting horizontal moments from the inertial and gravitational forces of the bipedal system vanish. In case the bipedal system does not slip and no other external forces than the ground reaction forces act on it the ZMP coincides with the *Center of Pressure (CoP)*. Maintaining the ZMP within the borders of a subset of the BoS has been used by various projects to control the walking motion of a humanoid robot (e.g., Sakagami et al., 2002; Wang et al., 2014). However, this approach leads to very conservative motions which do not resemble the dynamic appearance of human gait. In fact, human walking is characterized by ZMP locations very close to the borders of the actual BoS. In addition, the ZMP only reflects the current state of the system and does not provide any meaningful information to predict falls.

This work focuses on describing the human foot placement strategy in terms of the velocity-based *Capture Point (CaP)*, introduced by Pratt et al. (2006), Koolen et al. (2012), and Hof et al. (2005), since it treats gait phenomena as future events and provides a versatile method to predict and evaluate the gait of individual subjects. The Capture Point indicates the foot location which should be anticipated after a push to come to complete stop. It considers the minimum time required to perform a step as well as the step's maximum reachable distance and has been implemented in the gait control of humanoid robots in Englsberger et al. (2015), Koolen et al. (2013), and Krause et al. (2012) and described as a recovery strategy applied by humans as a response to unexpected perturbations in everyday situation by Aftab (2012). Furthermore, based on the Capture Point, strategies to adapt temporo-spatial gait parameters to varying environmental conditions and asymmetric step lengths in transtibial prosthetic gait have been associated with functional compensation strategies in order to reduce the risk of falling backwards in Hak et al. (2013a,b, 2014). Disregarding the maximum step length and the minimum step duration, the Capture Point is referred to as the *Instantaneous Capture Point (ICaP)*.

In addition, this work considers the *Angular Momentum* applied by the human walkers about the center of mass. It has been observed by Herr and Popovic (2008) that during straight and upright walking the average angular momentum about the principal axes remains close to zero. As discussed in Mombaur and Vallery (2018), the oscillations of the angular momentum around zero, even though small, are not small enough to be ignored and are contributing to the nature of walking, which is also in accordance with the observation of the virtual pivot point located above the center of mass (see Maus et al., 2010).

### 1.2. Contributions of this paper

In this work, we discuss different benchmarking criteria for their applicability to quantify the stability of bipedal locomotion. We propose a combined assessment of bipedal gait based on an extension of the Capture Point as well as the full body angular momentum as a benchmarking tool for human walking. The application of this method is demonstrated by computing data for two unimpaired subjects and one subject walking with a prosthesis.

We hypothesize that, in order to maintain a steadily stable walking motion, the desired foot location approached by unimpaired humans while moving the swing leg forward is correlated to the *Instantaneous Capture Point* (Pratt et al., 2006; Koolen et al., 2012). Furthermore, we conjecture that humans with deviating habitual gait patterns, asymmetric body properties or limb replacements aim at a similar stability strategy by adjusting their gait dynamics according to the modified dynamic and actuatory properties. This implies that a symmetric foot placement strategy is maintained by applying asymmetric gait dynamics. In order to analyze human gait for these strategies we reconstruct the dynamics of the human walking motion from motion data obtained in a gait laboratory. Introducing the *Residual Orbital Energy*, we are able to simultaneously analyze the reconstructed motions for the underlying whole-body dynamics as well as the foot placement strategy with respect to the Instantaneous Capture Point.

The application of the proposed method results in distinguishable gait dynamics for impaired and unimpaired humans with symmetric and asymmetric body proportions leading to the common objective, namely to move the swing leg toward the Instantaneous Capture Point. In order to validate the method, however, walking motions of many more subjects need to be investigated.

## 2. Methods

In this paper, we present a first study of the proposed benchmarking criteria. This includes their evaluation on existing walking data of a few subjects—two unimpaired subjects and one subject with a prosthesis—as a first indicator on the performance of the proposed method. A large statistical analysis of the benchmarking criteria is beyond the scope of this paper and will be subject of our future work.

The dynamics of the subjects' walking motion are reconstructed from recorded motion capture data using individualized multibody models of the subjects and optimal control methods in a least-squares sense. This approach ensures that the dynamics of the model are satisfied throughout the entire time horizon rather than only on discrete time steps. Based on the reconstructed motions, the whole-body dynamics as well as the temporo-spatial gait parameters are compared between the subjects. The analysis focuses on the behavior of the ICaP for each subject and, in particular, on the characteristics of each subject's foot placement with respect to the ICaP right at the *heel strike* event.

### 2.1. Motion recordings

The recordings include a full stride beginning with the *toe off* of the left foot of three subjects: (A) an unimpaired female subject, (B) an unimpaired male subject as well as (C) a male subject walking unilaterally with a transfemoral prosthesis on the right side. Some characteristics of the subjects are included in Table 1.

**Table 1:** Subject details.

	**Subject**	**Age [years]**	**Height [m]**	**Weight [kg]**	**Leg length [m]**
A	Female unimpaired	30	1.68	54.1	0.792
B	Male unimpaired	41	1.88	89.0	0.862
C	Male impaired	42	1.79	92.0	0.825

**Figure 1:**
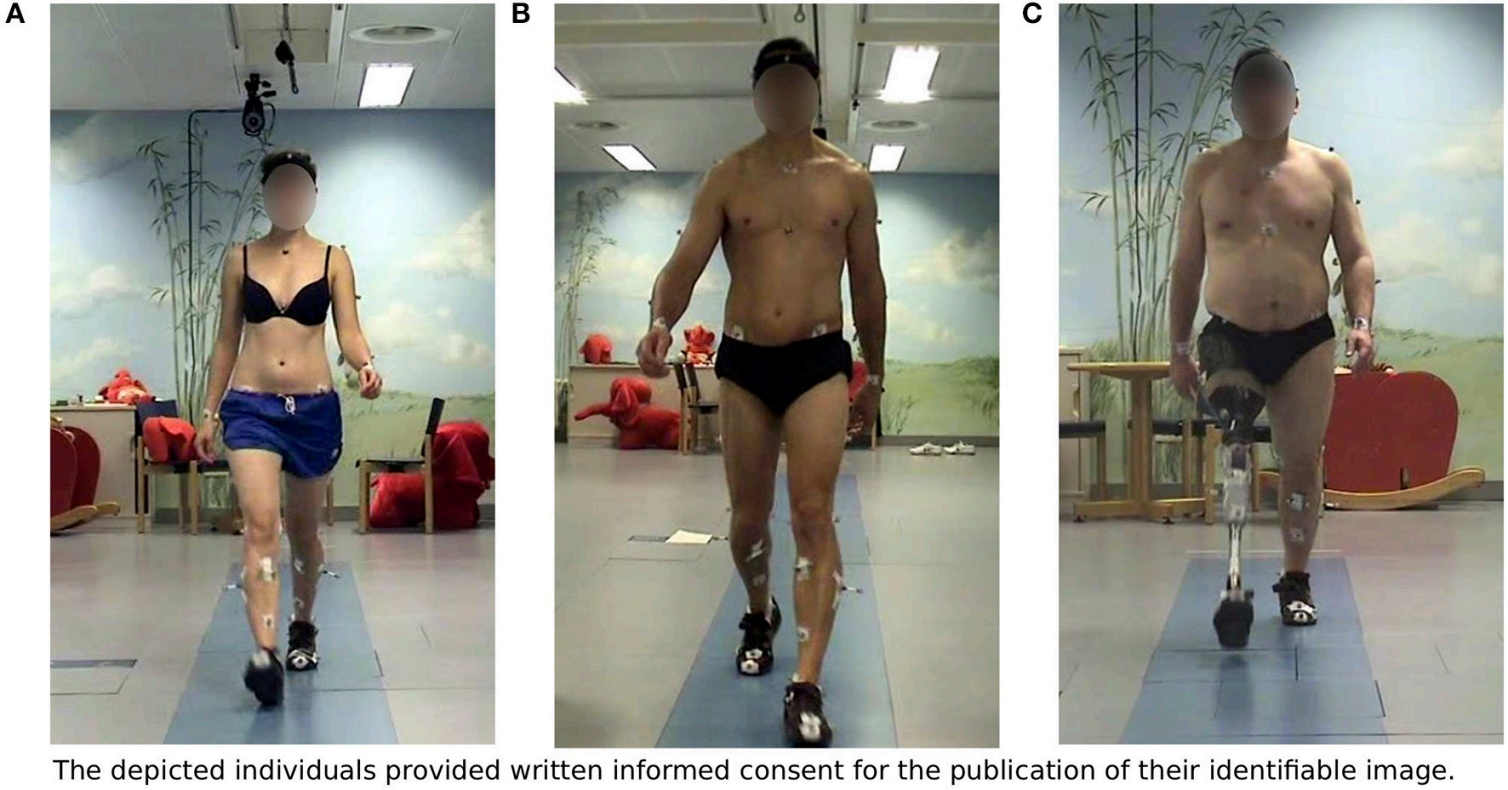
Subjects in the motion capture laboratory: **(A)** Unimpaired female, **(B)** Unimpaired male, **(C)** Male walking with a transfemoral prosthesis on the right side. All subjects gave written informed consent in accordance with the Declaration of Helsinki for the publication of their identifiable image.

The kinematic part of the walking motion of the subjects has been recorded using marker-based motion capturing. The recordings have been gathered in the *Clinic for Orthopedics and Trauma Surgery at Heidelberg University Hospital*^1^ located in the *Heidelberg University Orthopedic Hospital* equipped with a Vicon Motion Systems Ltd.^2^ MoCap system and three Kistler Instrumente GmbH^3^ force plates. An extended version of the Plug-In Gait marker set provided by Vicon Motion Systems Ltd. (2010) has been chosen to enable the recording of the full body motion for all subjects, as shown in Figure 1.

The subjects' gait has been recorded at self-selected walking speeds. The gait appearance of the unimpaired subjects can be considered healthy, symmetric and without any physical limitations. The impaired subject has been individually fitted with a prosthetic knee which also includes a customized socket and appropriately selected prosthetic components. This subject has been provided on the right side with the Össur hf. (2017a) Rheo prosthetic knee and the Össur hf. (2017b) Vari-Flex foot. His gait appears smooth and symmetric.

### 2.2. Models

The human body is modeled as a 13-segment multibody model with 34 degrees of freedom (DoFs). The rigid bodies represent the body segments *pelvis, left/right thigh, left/right shank, left/right foot, mid/upper trunk, left/right upper arm, left/right lower arm, left/right hand*, and *head*, respectively.

The rigid bodies are connected by the 3-DoF joints *right/left hip, right/left ankle, Lumbo-Sacral joint, right/left shoulder*, and *Cervicale* as well as the 1-DoF joints *right/left elbow* and *right/left knee*, respectively. The absolute translation and orientation of the entire system with respect to the global frame in *Euclidean* space is defined by the six DoFs for the absolute translation and orientation of the pelvis segment.

**Figure 2:**
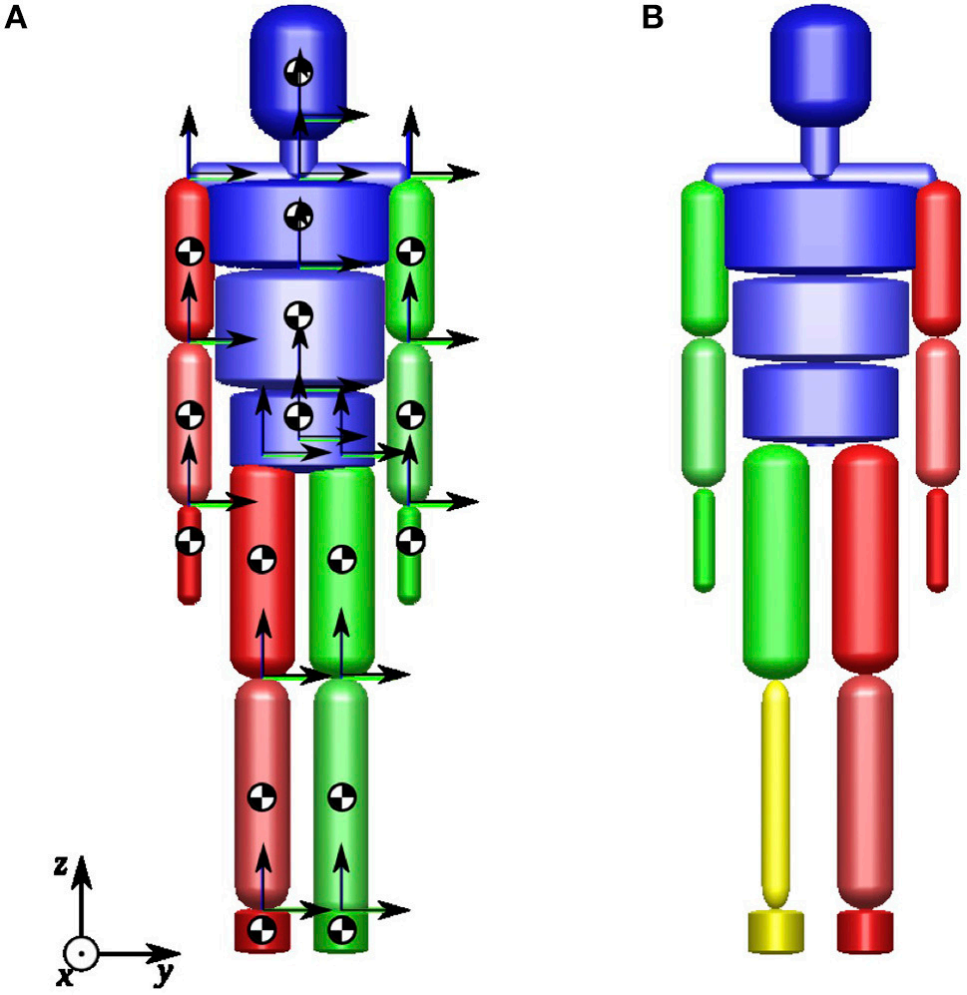
Multibody models of the full human body: **(A)** Full human body with segment COM positions and local coordinate frames, **(B)** Full human body with transfemoral prosthesis (yellow).

**Figure 3:**
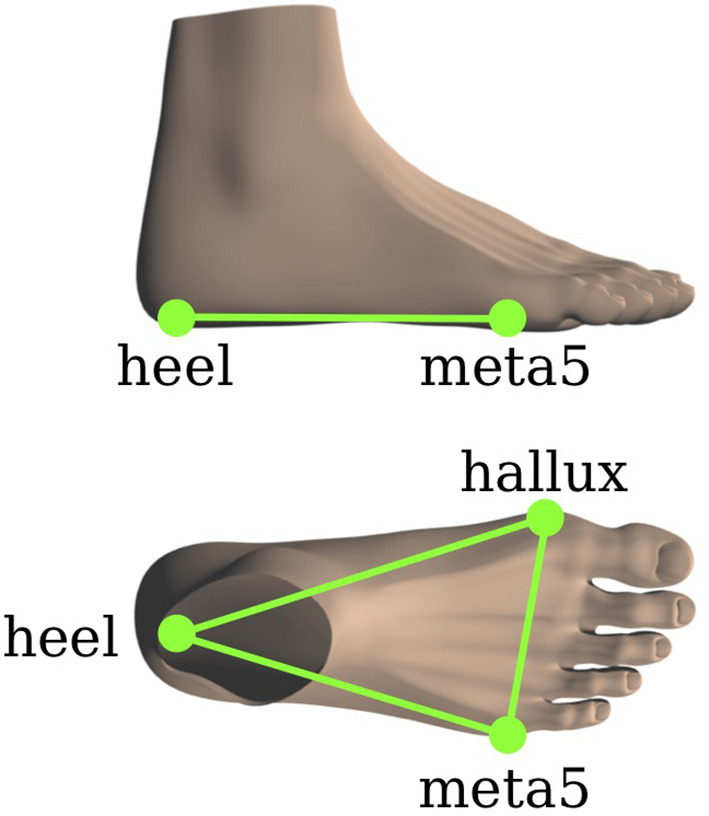
Foot model with the three contact points *hallux, meta5*, and *heel*.

The model is based on the 16-segment multibody model with 43 DoFs illustrated in Figure 2. During human walking, no significant motion occurs in the *Xiphoid* joint and the *right/left wrists*. Hence, zero DoFs are assumed between the middle and upper trunk as well as between the lower arms and the hands, respectively, and the model can be reduced to the model used in this study. The dynamic model parameters for all subjects were obtained using the regression equations provided by de Leva (1996). In addition, the dynamic model parameters for the prosthetic leg have been obtained by simple experiments involving scaling, balancing and oscillating the prosthesis. For Subject C the prosthetic leg's mass is approximately 35% the mass of his opposite leg. The model establishes ground contact with the feet which are represented by rigid triangular segments spanned by the three contact points *heel, hallux* and *meta5* as shown in Figure 3.

### 2.3. Equations of motion

Depending on the current gait phase, the mechanical system described above is subject to changing contact properties and can be described by a set of differential algebraic equations

(1) $$\dot{q}=v$$

(2) $$\dot{v}=a$$

(3) $$\left(\begin{array}{cc} H(q) & G{\left(q\right)}^{T}\\ G(q) & 0 \end{array}\right)\left(\begin{array}{c} a\\ -\lambda \end{array}\right)=\left(\begin{array}{c} -C(q,v)+\tau (q,v)\\ \gamma (q,v) \end{array}\right)$$

with the symmetric and positive-definite mass matrix ***H***, the generalized non-linear effects ***C***, the generalized internal forces ***τ***, the differential variables ***q*** and ***v*** for the positions and the velocities, respectively, the algebraic variables ***a*** and λ for the accelerations and the contact forces, respectively, as well as the *contact Hessian*

(4) $$\gamma (q,v)=-\dot{G}(q)v=-v^{T}\frac{\text{d}G(q)}{\text{d}q}v.$$

and additional constraints on position and velocity level

(5) g(q)=0

(6) $$G(q)\dot{q}=0$$

For non-redundant constraints ***g***(***q***) the contact Jacobian ***G***(***q***) has full rank and (3) can be uniquely solved.

Whenever the model gains contact with the ground, the perfectly rigid foot model causes discontinuous transitions from the generalized velocity ***v***^−^* before* the collision to the generalized velocity ***v***^+^* after* the collision. The transition is determined by

(7) $$\left(\begin{array}{cc} H(q) & G{\left(q\right)}^{T}\\ G(q) & 0 \end{array}\right)\left(\begin{array}{c} v^{+}\\ -\Lambda \end{array}\right)=\left(\begin{array}{c} H(q)v^{-}\\ 0 \end{array}\right)$$

where the first line determines the change of the system's momentum caused by the collision and **Λ** denotes the contact impulse.

### 2.4. Identification of walking motions by means of optimal control

In this paper, unimpaired and prosthetic human walking motions are reconstructed by fitting the motions of subject-specific dynamic models to motion capture data by formulating and solving a large-scale multi-phase *optimal control problem (OCP)* in a least-squares (LSQ) sense. In order to minimize the dimension of the optimal control problem the motion is reasonably fitted along the *generalized coordinates* of the model instead of the *Cartesian* coordinates.

The reference motions for the optimal control problems are created by converting the measured motion capture data from marker trajectories in Cartesian space into trajectories in joint angle space. This can be performed by approximating the motion of a subject-specific multibody model such that the distance between virtual markers defined on the model and the appropriate measured marker positions are minimized for each time frame considered. The fit is performed in a least-squares sense and considers the entire kinematic chain of the multibody model (see Sugihara, 2011; Felis, 2015). In this study, the reference motion has been fitted with an average matching error over all markers of 2.0 cm ± 1.1 (Female unimpaired), 2.0 cm ± 1.3 (Male unimpaired), and 1.6 cm ± 0.8 (Male impaired), respectively.

**Figure 4:**
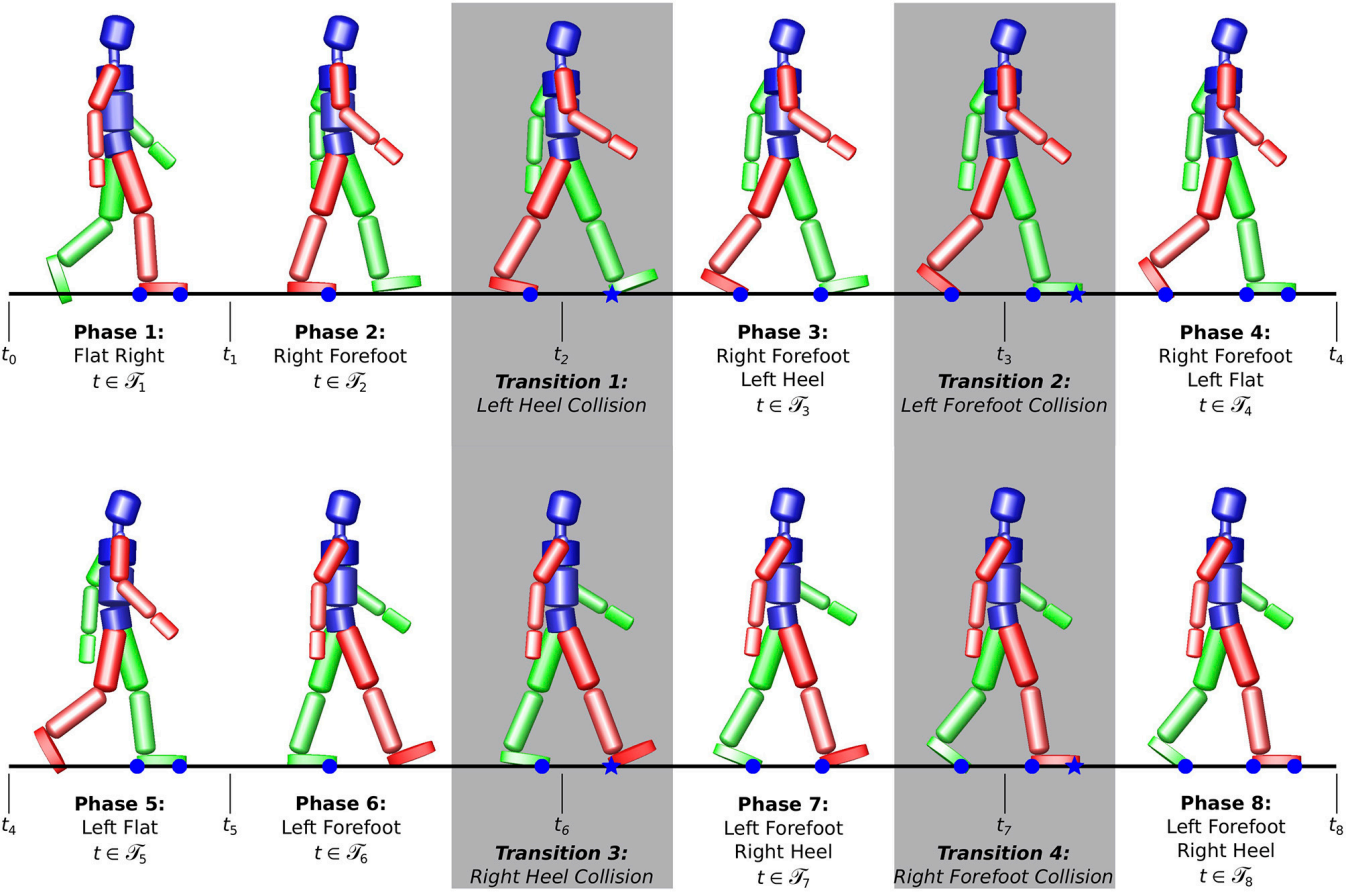
Gait phases for the optimal control problem. Transition phases (gray) are introduced to account for the discontinuities occurring to the velocities $\dot{q}$ at ground collision when a new contact is gained.

The optimal control problem is divided into 8 phases according to the phases of a whole gait cycle as well as four transitions to account for the discontinuities occuring when ground contact is established, see Figure 4. The gait phases can be distinguished by the different contact configurations between the human and the environment, expressed in the model as nonlinear point constraints, which determine the dynamics of the system.

The reference motions can be summarized in a set of time discrete postures expressed in terms of the generalized coordinates $q_{j}^{\text{ik}}\in {\mathbb{R}}^{n_{\text{dof}}},j=0,\ldots ,m$ at the time instances *t*_0_, …, *t*_*m*_∈ℝ. The least-squares problem is then described for the *n*_ph_ = 8 model stages by

(8) $$\underset{{}_{x(\cdot ),u(\cdot )}}{\min}\;\sum_{j=1}^{m}\;\frac{1}{2}||q_{j}^{\text{ik}}-q(t_{j})|{|}_{2}^{2}+\gamma_{u}||Wu(t_{j})|{|}_{2}^{2}$$

subject to:

(9) $$\dot{x}(t)=f_{i}(x(t),u(t)),$$

(10) $$x(t_{i}^{+})=h_{i}(x(t_{i}^{-})),$$

(11) $$0\leq g_{i}(t,x(t),u(t)),$$

(12) $$0=r^{\text{eq}}(x(0),\ldots ,x(T),u(0),\ldots ,u(T)),$$

(13) $$0\leq r^{\text{ineq}}(x(0),\ldots ,x(T),u(0),\ldots ,u(T)),$$

$$\text{for }t\in [\tau_{i-1},\tau_{i}],i=1,\ldots ,8,\tau_{0}=0,\tau_{8}=T$$

with the differential states

(14) $$x(t)=\left(\begin{array}{c} q(t)\\ \dot{q}(t) \end{array}\right)\in {\mathbb{R}}^{2n_{\text{dof}}}$$

and the model's generalized coordinates $q\left(t\right)\in {\mathbb{R}}^{n_{\text{dof}}}$, generalized velocities $\dot{q}\left(t\right)\in {\mathbb{R}}^{n_{\text{dof}}}$ and the controls $u\left(t\right)\in {\mathbb{R}}^{n_{\text{act}}}$ which represent the torques that act directly on the model's joints.

The first term of the objective function Equation (8) minimizes the sum of squared differences between the model joint angles ***q***(*t*)_*j*_ and the joint angles $q_{j}^{\text{ik}}$ from the inverse-kinematics analysis of the motion capture recordings. The second term regularizes the problem and accounts for the different magnitudes of the joint forces weighted by the diagonal matrix ***W*** = *diag*(*w*_*l*_), *w*_*l*_ > 0, *l* = 1…*n*_act_ and the factor *γ*_*u*_. The ODEs Equation (9) describe the model dynamics in each phase where the right hand sides $f_{i}:{\mathbb{R}}^{n_{x}}\times {\mathbb{R}}^{n_{u}}\times {\mathbb{R}}^{n_{x}}$ are characterized by the different constraint properties. Discontinuities in the generalized velocities $\dot{q}\left(t\right)$ that occur due to the model specific perfectly rigid impact at ground collision in case of touch-down events are handled using the phase transition functions Equation (10). Upper and lower bounds for the differential states ***x***(*t*) as well as the controls ***u***(*t*) are covered by the path constraints Equation (11). The path constraints for the generalized coordinates ***x***(*t*) are chosen to reflect the ranges for the typical walking motions. Additional constraints that, e.g., ensure physical feasibility such as unilateral ground contacts as well as switching conditions for phases are contained in the interior point constraints Equations (12, 13) which distinguish the several gait phases from each other.

Due to the hybrid dynamic character of the computed walking motions the optimal control problem is solved using the *direct multiple-shooting method* and a *piecewise linear control discretization*. The *multiple-shooting state parameterization* transforms the original boundary value problem into a set of initial value problems with corresponding continuity and boundary conditions. The multiple-shooting method is implemented in the software package MUSCOD-II (Bock and Plitt, 1983).

### 2.5. Angular momentum

Human walking is characterized by the alternating swinging motions of the upper and lower limbs. Counter-rotating motions of the upper and lower body are applied to balance the whole body angular momentum around the longitudinal axis and enable the human to walk straight. Oscillating angular momenta are applied in the frontal plane to facilitate the transfer of the body weight from one leg to the other. We analyze the angular momentum applied by the subjects in the horizontal plane (i.e., with respect to the vertical axis) by the motions of the upper and lower body for their contribution to the full body angular momentum in order to reveal individual strategies to compensate for asymmetric dynamic properties of the body or unbalanced habitual gait patterns in steady walking.

### 2.6. Foot placement

An intuitive approach to quantify and control stability in human walking is motivated as a response to an unexpected loss of balance, e.g., when a sudden perturbation occurs. Since walking can be considered a perpetual falling motion followed by a well-timed and well-placed step, we analyze the walking motion based on the *Capturability* concept introduced by Pratt et al. (2006), Hof et al. (2005) and Koolen et al. (2012) and the herein proposed location of the *Instantaneous Capture Point (ICaP)*

(15) $$r_{\text{icap}}=r_{\text{cop}}+\frac{{\dot{r}}_{\text{com}}}{\omega_{0}}.$$

with the current position of the *Center of Pressure ****r***_cop_, the velocity **ṙ**_com_ of the pendulum's mass and its *angular eigenfrequency* $\omega_{0}=\sqrt{g/l}$ with the pendulum's length *l*. In addition, we study the *Orbital Energy* *E*_lip_ of the *Linear Inverted Pendulum Model (LIPM)* underlying the walking system which we normalize by dividing the quantities related to positions and lengths by each subject's leg length (hip to ankle) such that

(16) $$\begin{array}{l} {E^{\prime }}_{\text{lip}}=\frac{1}{2}{\dot{r}}_{\text{com}}^{\prime 2}-\frac{1}{2}{\left({r^{\prime }}_{\text{com}}-{r^{\prime }}_{\text{cop}}\right)}^{2}\omega_{0}^{2}. \end{array}$$

We analyze the normalized orbital energy $E_{\text{lip}}^{\prime }$ of the LIPM at a given time instance taking into account the current COM height and assuming that the velocity vector is horizontal. In particular, we are interested in $E_{\text{lip}}^{\prime }$ at the time instances right after the heel strike and propose the expression *Residual Orbital Energy* $E_{\text{res}}^{\prime }$. We use this parameter to characterize the specific gait pattern of the subject established by the self-selected combination of the step length, the step width and the magnitude of the ground collision impact at heel strike which causes a loss of kinetic energy and, thus, gait velocity. The link between the gait velocity and the Orbital Energy is established in the first term of Equation (16) and included into the model by Equation (10).

## 3. Results and discussion

### 3.1. Angular momentum and foot placement

**Figure 5:**
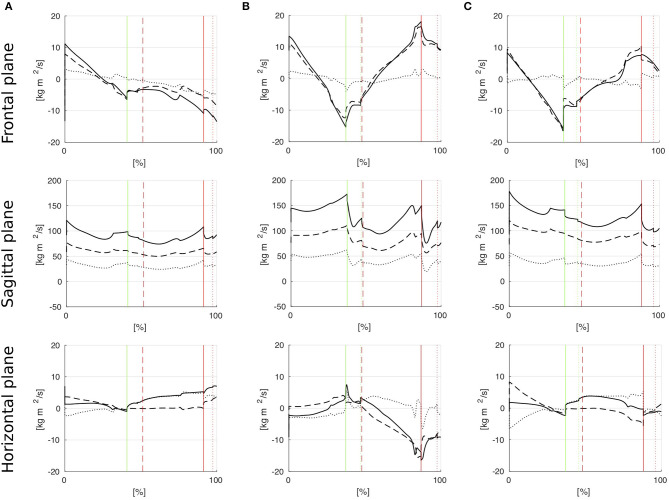
Angular momenta in frontal, sagittal, and horizontal plane for the upper body (dashed), the lower body (dotted) and the full body (solid) motion. Vertical lines indicate the gait events *heel strike* (solid), *toe strike* (dotted) and *toe off* (dashed) for the left (green) and right (red) feet, respectively. **(A)** Unimpaired female, **(B)** Unimpaired male, **(C)** Male with prosthesis.

**Figure 6:**
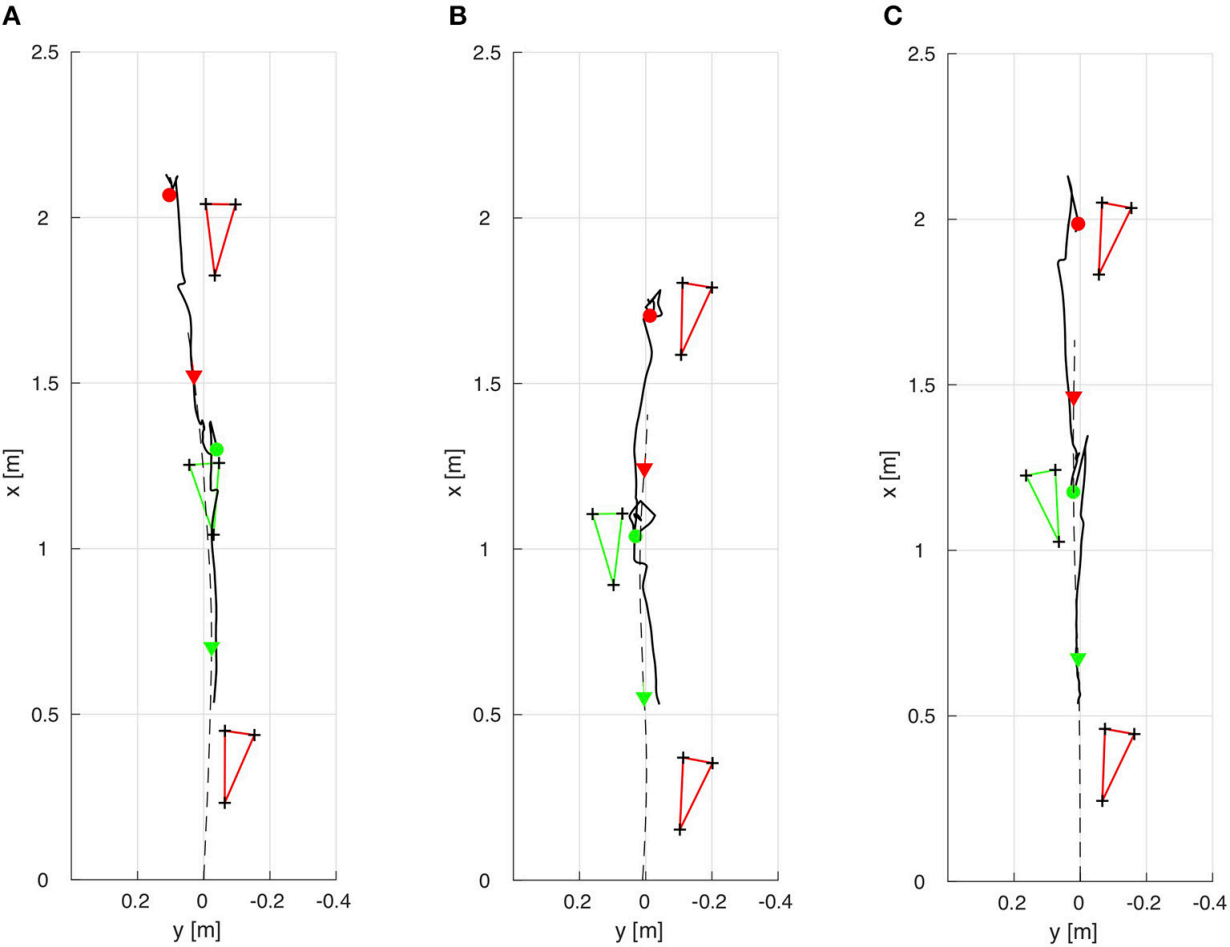
Footprints of the right (red) and left (green) feet as well as the trajectories of the ICaP (solid) and the ground projection of the COM (dashed) in the *xy*-plane. The ICaP positions (˙) are illustrated right at the heel strike of the right and left feet, respectively, along with the COM positions (▿) at the same time instances. **(A)** Unimpaired female, **(B)** Unimpaired male, **(C)** Male with prosthesis.

The results for the angular momentum applied by the subjects in the upper body, lower body and full body, respectively, in the frontal, sagittal, and horizontal planes are shown in Figure 5. Figure 6 shows that the subjects choose their step locations such that the ICaP is approximately reached by the forefoot to midfoot of the anterior foot in sagittal direction. The subjects maintain a well-balanced gait in terms of the ICaP being smoothly moved between both feet in lateral direction to facilitate lateral oscillation from one stance leg to the other.

Subject A has balanced momenta in the frontal and horizontal plane during the left swing phase (Figure 5A). However, she diverges her momenta in both planes during the right swing phase. During that phase, the upper body has no significant contribution to the full body momentum to balance the momentum applied by the lower body. This causes Subject A to experience a strong tilt and slight turn to the left. She adjusts her step location accordingly to follow the ICaP by placing her swing foot further into the same direction, see Figure 5A.

The angular momentum of Subject B in the frontal plane is regular and symmetric mostly established by a balanced pendulum motion of the upper body (Figure 5B). The angular momentum in the horizontal plane can be considered strongly unbalanced in the right swing phase. This leads to a stronger external rotation of the right foot compared to the left foot rotation (Figure 6B). However, since the greater amount of angular momentum is applied by the upper body, balancing the full body momentum can be achieved by exploiting friction between the stance foot and the ground. Applying this strategy, Subject B achieves a perfectly straight walking path.

Subject C shows well-balanced angular momenta in the frontal and horizontal plane in the time instances right before the heel strike events of both feet (Figure 5C). Although walking with a transfemoral prosthesis on his right side, he adjusts his upper body motions to establish strongly asymmetric angular momenta which compensate for the equally asymmetric momenta from the lower body. Applying this strategy, Subject C achieves a well-balanced and straight walking path, see Figure 6C.

The angular momenta in the sagittal plane have typical profiles for all subjects. The whole body momentum is created by forward swinging motions of the arms and legs and is partially dissipated at heel strike.

### 3.2. Residual orbital energy

The normalized residual orbital energy $E_{\text{res}}^{\prime }$ of each subject right at the heel strike events as well as their average values are summarized in Table 2. As can be seen in Equation (16), a greater value for $E_{\text{res}}^{\prime }$ is caused either by greater gait velocities, greater CoP-ICaP distances, or both. In these cases, capturing from sudden disturbances become increasingly difficult. Accordingly, lower values for $E_{\text{res}}^{\prime }$ are caused by lower gait velocities, shorter CoP-ICaP distances, or both and capturing from disturbances becomes less difficult.

**Table 2:** Normalized residual orbital energy $E_{\text{res}}^{\prime }\left[1/s^{2}\right]$ at left and right hell strike (HS) and the average value.

	**Subject**	**Left HS**	**Right HS**	**Average**
A	Unimpaired female	0.0664	0.0660	0.0662
B	Unimpaired male	0.0783	0.0435	0.0609
C	Male with prosthesis	0.0723	0.0649	0.0686

Considering the Residual Orbital Energy $E_{\text{res}}^{\prime }$, the values in Table 2 reveal a symmetric behavior for Subject A. In contrast, Subject B shows a strongly asymmetric behavior mostly caused by an asymmetric application of upper body angular momentum (Figure 5B) and, thus, an asymmetric gait velocity right at the heel strike events. The residual orbital energy $E_{\text{res}}^{\prime }$ at the heel strike event of the right (prosthetic) foot of Subject C is slightly less than at left heel strike. We might assume that Subject C reduces his gait velocity and, thus, his impact at ground collision in order to prevent pain at the socket-stump interface.

Using the Residual Orbital Energy $E_{\text{res}}^{\prime }$ in combination with the foot placement strategies with respect to the ICaP as a benchmarking tool, we consider the individual walking motions of the Subjects A and B (both unimpaired) as irregular gait and the walking motions of Subject C (walking with a prosthesis) as conscious gait.

## 4. Conclusion

In this work, we have investigated benchmarking criteria that help to quantitatively assess the stability of walking motions. As a first test, we have applied them to the walking motions of two unimpaired subjects and one subject walking with a transfemoral prosthesis which have been reconstructed from motion capture recordings using multibody dynamics and optimal control methods. The reconstructed walking motions have been analyzed for their dynamics and findings are gathered on how unbalanced habitual gait patterns can lead to irregular walking motions. On the other hand, the analysis provides insights into the individual strategies applied by the subject walking with the prosthesis to compensate for his asymmetric dynamic properties of the lower limbs and achieve a perfectly balanced walking motion.

For all subjects, the ICaP is shown to be consistently approached by the swing foot even if the walking paths deviate in lateral direction. The subjects choose their step locations such that the ICaP is located in anterior-medial direction of the foot and maintain a Residual Orbital Energy at heel strike >0 in order to facilitate the forward propulsion and lateral oscillation characteristic for human walking. The Residual Orbital Energy combines the subject's distance maintained between the CoP and the ICaP at each step with the gait velocity which, in human walking, is controlled to a great part by the rate of change of angular momentum.

In our proposed method we simultaneously interpret foot placement with respect to the ICaP as well as the Residual Orbital Energy. By deconstructing the walking motion into these parts we are able to reveal hidden phenomena in gait which superficially appears regular and symmetric. Regarding these criteria, the walking motion of both unimpaired subjects turn out to be irregular while the impaired subject's gait is well-controlled and well-balanced. We suspect that the unimpaired subjects' awareness of their physical capabilities provides them with enough confidence to allow for less conscious gait. On the other hand, we suspect that the impaired subject is aware of his limited ability to control his prosthetic leg and, therefore, follows a more cautious and conscious approach to walking.

The results are encouraging, but the criteria obviously remain to be further tested and validated on large sets of data. A thorough evaluation of these criteria based on existing whole-body walking data (i.e., including upper body and arms), e.g., from data bases, KoroiBot Motion Capture Database^4^, or newly collected whole-body data for unimpaired subjects and subjects walking with prostheses will be conducted. It remains to be determined how subject-specific properties have to be systematically taken into account to adjust the proposed criteria and allow for appropriate classifications.

## Ethics statement

This study was carried out in accordance with the recommendations of the ethics guidelines 158/2006 issued by the ethics committee of the University of Heidelberg with written informed consent from all subjects. All subjects gave written informed consent in accordance with the Declaration of Helsinki. The protocol was approved by the ethics committee of the University of Heidelberg.

## Author contributions

K-LH developed the hypotheses, developed and executed the procedure for data processing and analysis, and wrote the manuscript. SW designed the experiment, collected data and advised in data analysis, KM advised in data processing and analysis and contributed to writing the manuscript.

### Conflict of interest statement

The authors declare that the research was conducted in the absence of any commercial or financial relationships that could be construed as a potential conflict of interest.

## Footnotes

^1^http://www.vicon.com

^2^http://www.kistler.com

^3^Heidelberg MotionLab, http://www.heidel-motionlab.de

^4^KoroiBot Motion Capture Database, 2016.
